# Electroconvulsive therapy and structural neuroplasticity in neocortical, limbic and paralimbic cortex

**DOI:** 10.1038/tp.2016.102

**Published:** 2016-06-07

**Authors:** T Pirnia, S H Joshi, A M Leaver, M Vasavada, S Njau, R P Woods, R Espinoza, K L Narr

**Affiliations:** 1 Department of Neurology, Ahamason-Lovelace Brain Mapping Center, University of California Los Angeles, Los Angeles, CA, USA; 2 Department of Psychiatry and Biobehavioral Sciences, University of California Los Angeles, Los Angeles, CA, USA

## Abstract

Electroconvulsive therapy (ECT) is a highly effective and rapidly acting treatment for severe depression. To understand the biological bases of therapeutic response, we examined variations in cortical thickness from magnetic resonance imaging (MRI) data in 29 patients scanned at three time points during an ECT treatment index series and in 29 controls at two time points. Changes in thickness across time and with symptom improvement were evaluated at high spatial resolution across the cortex and within discrete cortical regions of interest. Patients showed increased thickness over the course of ECT in the bilateral anterior cingulate cortex (ACC), inferior and superior temporal, parahippocampal, entorhinal and fusiform cortex and in distributed prefrontal areas. No changes across time occurred in controls. In temporal and fusiform regions showing significant ECT effects, thickness differed between patients and controls at baseline and change in thickness related to therapeutic response in patients. In the ACC, these relationships occurred in treatment responders only, and thickness measured soon after treatment initiation predicted the overall ECT response. ECT leads to widespread neuroplasticity in neocortical, limbic and paralimbic regions and changes relate to the extent of antidepressant response. Variations in ACC thickness, which discriminate treatment responders and predict response early in the course of ECT, may represent a biomarker of overall clinical outcome. Because post-mortem studies show focal reductions in glial density and neuronal size in patients with severe depression, ECT-related increases in thickness may be attributable to neuroplastic processes affecting the size and/or density of neurons and glia and their connections.

## Introduction

The high rates of major depression, affecting an estimated 350 million people worldwide,^1^ and the only moderate success rates of available antidepressant treatments underscore the importance of unraveling the biological bases of therapeutic response. In the largest federally sponsored clinical medication trial conducted to date, up to one-third of patients failed to sufficiently benefit from two or more standard pharmacotherapies.^2^ For such patients, who are described to have treatment-resistant depression, electroconvulsive therapy (ECT) remains the most effective treatment option, with response rates exceeding 70%.^3, 4, 5^ Contrasted with standard pharmacotherapy, which can take 2 months or longer to work, a full remission of symptoms can occur within 2–4 weeks with ECT.^6^ However, similar to pharmacotherapy, the success of ECT is variable and the mechanisms underlying symptom improvement remain unclear. Identifying biomarkers of ECT response may clarify the mechanisms of antidepressant action and promote more effective individually tailored treatments for patients with severe depression.

In animal models of ECT, repeated electroconvulsive shock (ECS) elicits widespread cell proliferation in both rodents and non-human primates. Whereas neurogenesis is observed in the hippocampus, gliogenesis and pronounced volumetric changes are also found in additional areas including the amygdala and prefrontal cortex after ECS.^7, 8, 9, 10, 11, 12, 13^ Recently, molecular markers of adult neurogenesis have also been reported in frontal areas in days to weeks after ECS.^14^ Although studies using pharmacological interventions induce similar treatment-related cell proliferation, these effects appear less pronounced in comparison with ECT.^14^ Post-mortem studies further demonstrate that depressed patients have reduced glial cell density and neuronal size compared with healthy volunteers in prefrontal regions including the anterior cingulate cortex (ACC), and connected dorsolateral (DLPFC) and ventral (VPFC) prefrontal cortices.^15, 16, 17^ Correspondingly, neuroimaging studies report an altered brain structure in depression, including reductions in the cortical gray matter;^18, 19, 20^ alterations in structure of the ACC, DLPFC, VPFC and ventromedial prefrontal cortex are frequently linked with the disorder.^21, 22, 23^ Taken together, these data support a neurotrophic model of depression and antidepressant response, suggesting that ECT may elicit structural plasticity within prefrontal and limbic regions.

To date, structural imaging studies in patients receiving ECT have largely focused on subcortical regions such as the hippocampus and amygdala because of their potential links with adult neurogenesis occurring in the hippocampal dentate gyrus.^24, 25^ Several groups have demonstrated that increases in the hippocampal and amygdala volume occur with ECT,^26, 27, 28, 29^ and relationships with symptom improvement have been observed.^30^ Evidence demonstrating ECT-related structural plasticity in other cortical areas involved in the neural circuitry governing mood and emotion is more sparse. For example, a prior study showed cortical gray matter changes with ECT in the DLPFC and VPFC cortex, but used a region-of-interest (ROI) approach rather than a cortex-wide analysis.^27^ Another study reported ECT-related gray matter change in the subgenual ACC and prefrontal cortex, although only 10 ECT patients were investigated.^31^ Neither of these studies detected associations with symptom improvement, perhaps as a limitation of sample size. At least one study failed to show changes in the gray matter volume in cortical or subcortical limbic regions, with ECT using voxel-based morphometry methods.^32^

To our knowledge, no published study has addressed whether regional reductions in cortical thickness, a measure that may be more sensitive to alterations in the glial and neuronal microstructure than volume,^33, 34^ are associated with or predict clinical response to ECT. The current investigation prospectively followed patients with treatment-resistant depression during an ECT treatment index series. We compared cortical thickness measured at high spatial resolution across the entire cortical mantle as well as across 33 anatomically defined cortical regions at three time points. The effect of diagnosis as well as normative values and variance were established through comparisons between patients scanned at baseline and controls scanned at two time points. For regions showing ECT effects, ROI analyses were used to address relationships and predictors of clinical response.

## Materials and methods

### Participants

Patients were recruited from individuals scheduled to begin ECT treatment at the Resnick Neuropsychiatric Hospital, University of California, Los Angeles (UCLA). Two prior studies have investigated an overlapping, although not identical, sample of patients to investigate changes in hippocampal morphometry and functional connectivity.^30, 35^ Eligibility criteria included a diagnosis of recurrent depression and a current depressive episode lasting 6 months or greater. Patient diagnoses were established by clinical consultation using Diagnostic and Statistical Manual of Mental Disorders IV-TR^36^ criteria and confirmed using the Mini-International Neuropsychiatric Interview.^37^ Patients with independently diagnosed comorbid psychiatric disorders including schizophrenia, schizoaffective disorders, post-traumatic stress disorder, attention hyperactive deficit disorder, dissociative disorders and patients separately diagnosed with anxiety disorders were excluded from the study. Bipolar disorder was not exclusionary, although patients in the manic phase of illness were not eligible for participation. Other exclusion criteria included comorbidity of dementia, first episode of depression, late onset of depression (>50 years of age), depression related to a medical condition or ECT or other neuromodulation therapies in the previous 6 months. All patients were tapered off psychotropic medications including antidepressants and benzodiazepines and with complete cessation of these medications for at least 48–72 h before initiation of ECT treatment.

Demographically similar healthy controls were recruited from the Los Angeles area using advertisements and screened with the M.I.N.I. to exclude a history of depression, other psychiatric illness, as well as antidepressant use. Additional exclusion criteria for all subjects included a history of alcohol or substance abuse within 6 months and/or dependence within 12 months of participation, neurological disorders or condition contraindicated for magnetic resonance imaging (MRI).

Of a total of 41 patients who enrolled in the study, 29 (18 females and 11 males) completed all three study time points (Table 1). Of the 12 patients who failed to complete all time points, 2 continued ECT elsewhere and 3 discontinued ECT early. Attrition for the remaining patients was due to inability to come in for or to tolerate repeat scanning or due to scanner hardware failures. Patients who failed to complete study procedures did not differ from those who completed all study time points in terms of age, sex, weight, ethnicity, education or dextrality (all *P*>0.05). Mood scales (Hamilton Rating Scale for Depression (HAM-D) and Montgomery–Åsberg Depression Rating Scale) at study entry also did not differ between completers and non-completers (*P*>0.05). The sample also included 29 controls (16 females and 13 males; Table 1). All participants provided written informed consent for participation as approved by the UCLA Institutional Review Board.

### Data acquisition

**Figure 1:**
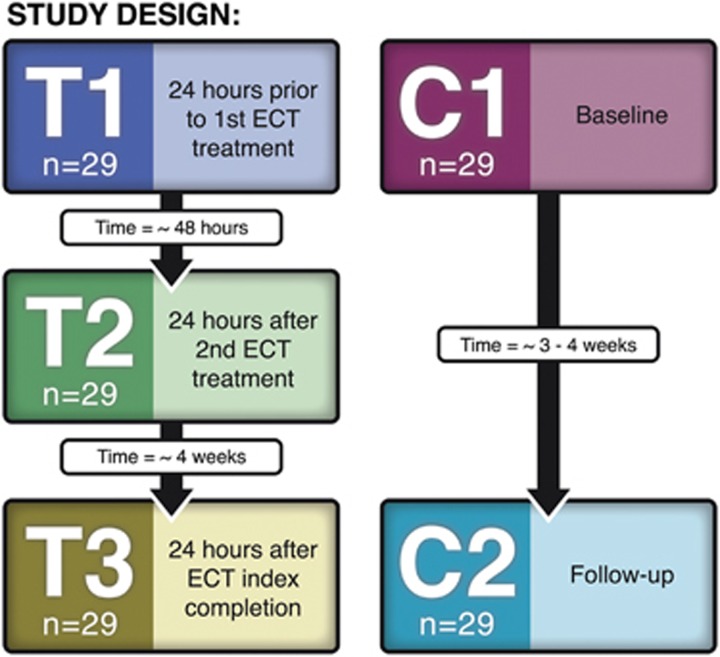
Study design. ECT, electroconvulsive therapy.

Patients received clinical assessments and structural scanning at three time points: T1 (baseline) completed within 24 h before the first ECT treatment; T2 completed within 48 h after their second ECT treatment; and T3 completed within 1 week of completing the ECT treatment index series. Controls completed scans at two time points with intervals equivalent to patients' T1 and T3 assessments (Figure 1).

### Clinical assessments

The Hamilton 17-item (HAM-D^39^) and Montgomery-Åsberg^40^ Depression Rating Scales were administered at each time point to assess symptoms and treatment response. As these scales are highly correlated, HAM-D ratings were chosen as the primary measure of clinical response. For descriptive purposes, other clinical measures including lifetime illness and length of current episode were also recorded. Clinical information is provided in Table 1.

### ECT treatment

ECT (5000Q MECTA, Tualatin, OR, USA) was administered three times a week, using a standard protocol for anesthesia (methohexital at 1 mg kg^−1^ dosage) and paralysis (succinylcholine at 1 mg kg^−1^ dosage). ECT followed the seizure threshold (ST) titration method where after establishing the ST, treatments were delivered at 5 to 6 × ST for right unilateral (RUL) d'Elia lead placement, using an ultrabrief pulse width (0.3 ms). The majority of patients (80%) received RUL exclusively. On the basis of clinical decisions independent of the study protocol, some patients were switched to bilateral placement during the course of the index series. Bilateral ECT was delivered at 1.5 × ST using a brief pulse width (0.5 ms). The length of ECT treatment index was clinically and individually determined, where patients received a mean of 11.2 ECT treatments (Table 1). ECT is known to produce large clinical effects. Assuming that biological effects might be of lesser magnitude, the current study was designed to detect within-subject differences of Cohen's *d*=0.53 (medium effect), with 80% power, two-tailed alpha of 0.05.

### MRI acquisition and analysis

High-resolution T1-weighted structural scans were acquired on a Siemens 3 T Allegra system (Erlangen, Germany) using a three-dimensional echo-planar imaging-navigated multiecho MPRAGE sequence optimized for morphometric analysis with real-time motion correction^41^ (echo time/repetition time=1.74, 3.6, 5.46, 7.32/2530 ms, inversion time=1260 ms, flip angle=7°, field of view=256 × 256 mm, 192 sagittal slices, voxel resolution=1.3 × 1.0 × 1.0 mm^3^).

Structural images were processed using the FreeSurfer's image analysis suite (5.3.0, http://surfer.nmr.mgh.harvard.edu/). Processing steps included correction for magnetic field inhomogeneities, removal of non-brain tissue, polygonal tessellation of the white/gray matter and pial cortical surfaces and parcellation of the cortical mantle into distinct cortical units leveraging local curvature and other contextual information.^42, 43^ For within-subject analysis, the FreeSurfer's longitudinal processing stream created an unbiased within-subject template across time points using robust, inverse consistent registration,^44^ from which surface tessellation and parcellations were initialized. Each image was visually inspected, and topographical errors in surface extraction were corrected. Cortical thickness was measured using the shortest distance between the white–gray and pial interface at each vertex and projected to the tessellated pial surface smoothed using a 10-mm full-width at half maximum kernel. Methods for cortical thickness calculation have been validated against histological analysis^45^ and manual measurements.^46^ Notably, the use of volumetric navigators for prospective motion correction^41^ and the longitudinal pipeline for cortical thickness analysis served to reduce the potential for motion-related artifacts.

### Statistical analysis

#### Effects of ECT

Vertex-wise changes in the cortical thickness across time within patient and control groups were separately evaluated using a general linear mixed model, with time as a fixed factor and subject as a random factor. For patients, subsequent *t*-tests were used to compare time points pairwise (that is, between T1 and T2, T2 and T3, and T1 and T3) using UCLA Shape Tools and Rshape^47^ (www.bmap.ucla.edu). A false discovery rate (FDR) threshold of *q*=0.05 controlled for multiple comparisons made across the cortex.

To increase signal-to-noise by searching functionally homologous regions, and to allow for the simultaneous assessment of lateralized effects, cortical thickness was also averaged within 33 ROIs defined anatomically from the Desikan–Killiany Atlas during the Freesurfer segmentation procedures. Analysis of cortical ROI thickness using the statistical model described above with the addition of hemisphere as a within-subject variable was implemented in SPSS (IBM SPSS Statistics for Windows, Version 22.0, Armonk, NY, USA). An FDR of *q*=0.05 was used as the threshold of significance for the multiple cortical ROIs examined. Follow-up comparisons addressed changes between the three time points pairwise. In *post hoc* analyses, these same analyses included lead placement, defined as the % of ECT sessions including RUL, as an additional covariate to determine the influence of lead placement on the observed ECT effects.

#### Effects of clinical response and predictive effects

Relationships with HAM-D scores were examined only in cortical ROIs showing significant overall effects of ECT, where a more liberal threshold of *P*<0.05 was used as the criterion for significance. Here, the general linear mixed model determined whether changes in the cortical thickness varied in treatment responders and non-responders where clinical response was defined as a >50% improvement in HAM-D scores over the course of ECT. For these models, the subject was again included as a random factor, response status and HAM-D scores were included as factors and hemisphere was included as a repeated measure.

For the same ROIs, the general linear model (GLM) examined whether variations in cortical thickness measured at baseline and early in the course of ECT (at T1 and T2, respectively) predicted overall clinical response, defined as the extent of change in HAM-D scores by subtracting scores between T1 and T3. As these analyses were cross-sectional, age and sex were included as covariates.

#### Cross-sectional effects of diagnosis

Finally, regions showing ECT effects were then also compared between patients and controls at baseline to establish main effects of diagnosis and determine whether cortical thickness values showing ECT effects normalize with treatment. As for the analyses of correlations with clinical response and predictive effects, these analyses were performed only in regions showing overall ECT effects where *P*<0.05 was used to determine significance. A summary of the statistical analyses performed is provided in Supplementary Table 1.

## Results

### Demographic and clinical variables

Patients and controls were similar in distribution of age, F(1, 57)=0.06, *P=*0.80, sex, *χ*^2^(1, 57)=0.51, *P*=0.47, and dextrality *χ*2(1, 57)=0.015, *P=*0.90 (Table 1).

HAM-D ratings improved significantly (reduction in score) with ECT, F(2, 28)=36.65, *P*<0.0001 (Supplementary Table 2). Using a threshold of 50% symptom improvement at the end of the treatment index, 62% (*n*=18) of patients were categorized as responders.

### Effects of ECT

**Figure 2:**
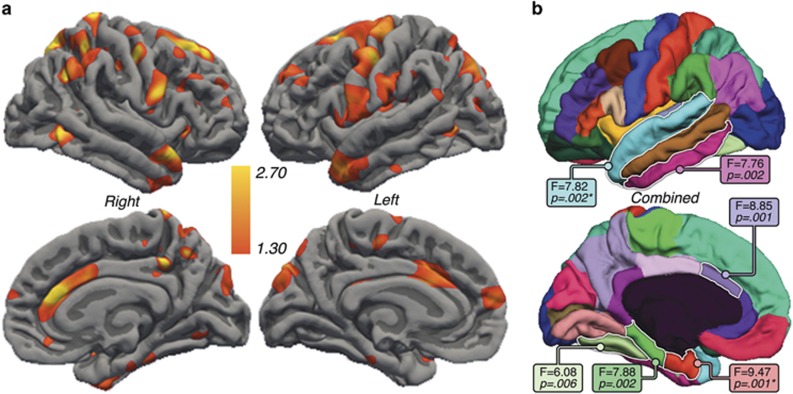
(**a**) Significant differences in the cortical thickness with electroconvulsive therapy (ECT) assessed linearly between baseline, T2 and T3 are indicated in hot colors (false discovery rate (FDR) thresholded at *q*=0.05). (**b**) Cortical regions of interest showing F- and *P*-values for regions showing significant ECT effects. Regions with an asterisk indicate significant ECT by hemisphere effects.

**Figure 3:**
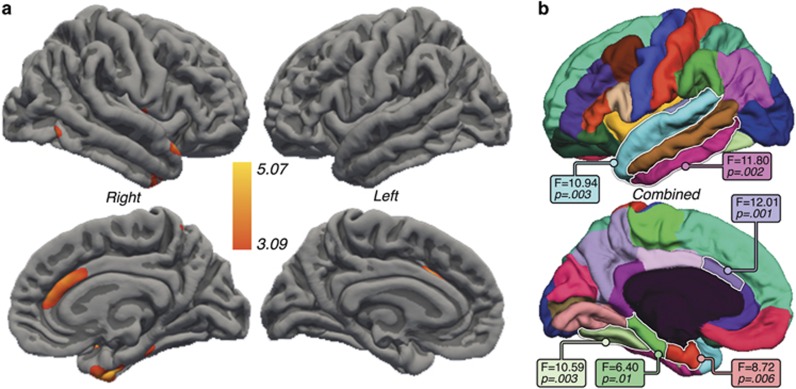
(**a**) Significant differences in cortical thickness between baseline and the end of the electroconvulsive therapy (ECT) treatment series examined pairwise between T1 and T3 are shown in hot colors (false discovery rate (FDR) thresholded at *q*=0.05). (**b**) Cortical regions of interest showing F- and *P*-values for regions showing significant change in thickness between T1 and T3.

General linear mixed model analysis of the entire cortex showed significant main effects of ECT in the bilateral ACC, superior temporal gyrus, temporal pole, parahippocampal gyrus and in clusters across the prefrontal, parietal association and motor cortex after FDR correction (Figure 2a). Pairwise comparisons of time points showed significant changes in the bilateral ACC, right parahippocampal gyrus, superior temporal gyrus and temporal pole (FDR-corrected, *P=*0.05) between T1 and T3 (Figure 3a). All changes in the cortical thickness over time represented increases in thickness in patients, and no regions showed reductions with ECT. Controls exhibited no significant regional changes in cortical thickness across time.

**Figure 4:**
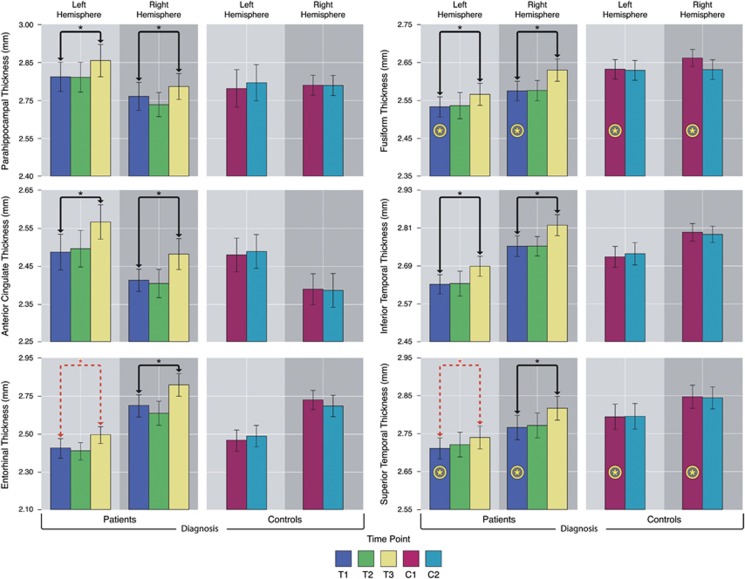
Graphs showing the mean thickness for regions of interest showing significant electroconvulsive therapy (ECT) effects plotted across time and in patients and controls. Black bars indicate significant effects of ECT across hemispheres. Dashed red bars indicate the presence of ECT by hemisphere interactions and indicate subthreshold results within the hemisphere. Yellow stars indicate significant effects of diagnosis, that is, between patients and controls, at baseline.

For ROI analysis, a corrected alpha level of 0.0088 was used as the threshold for significance corresponding to an FDR of *q*=0.05. Distributions of ROI thickness did not deviate from normality (Kolmogorov–Smirnov test, all *P*>0.20). Significant effects of ECT were observed in six cortical regions: the ACC, parahippocampal, entorhinal, superior temporal, inferior temporal and fusiform cortex (Figure 2b). Significant hemispheric interactions were observed in the entorhinal cortex and superior temporal gyrus, where significant effects of ECT were observed in the right hemisphere (F(1,28)=9.31, *P=*0.0001 and F(1,28)=7.02, *P=*0.003, respectively); trends were observed in the left hemisphere (*P=*0.07 and *P=*0.057). Pairwise comparison of T1 and T3 showed effects in the same cortical regions (Figure 3b). Follow-up analysis including the percentage of RUL placement as a covariate did not alter the results, with the exception that interactions with hemisphere were no longer significant for any cortical ROI. Figure 4 shows change over time for each ROI, which were found to show ECT effects, in patients and controls; Supplementary Table 3 provides the mean for all cortical ROIs.

### Cross-sectional effects of diagnosis

To determine whether ECT promotes normalization toward control values, differences between patients and controls were examined at baseline across the cortex and in cortical ROIs showing significant ECT effects. Cortex-wide analyses did not survive FDR thresholding at *q*=0.05 in any region. However, within cortical ROIs, patients showed reduced thickness in the fusiform, F(1, 54)=9.01, *P=*0.004 and the superior temporal cortex F(1, 54)=4.59, *P=*0.037 compared with controls (Figure 4; Supplementary Table 4).

### Effects of clinical response

**Figure 5:**
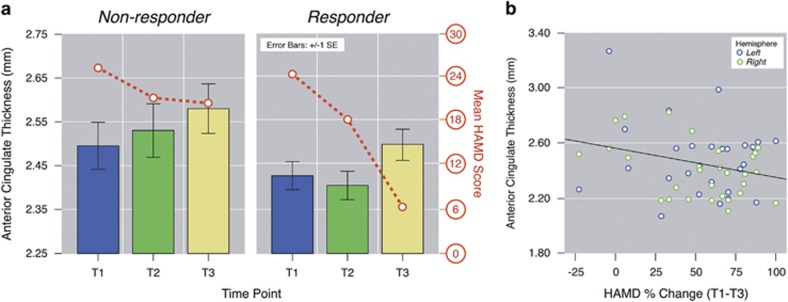
(**a**) Significant interactions between anterior cingulate cortex (ACC) thickness and the Hamilton Rating Scale for Depression (HAM-D) score (red line) across each time point in responders (right) and non-responders (left), (**b**) significant relationships between ACC thickness measured at T2 and change in HAM-D scores between baseline and the end of the electroconvulsive therapy (ECT) index (T1–T3).

Significant correlations between change in the cortical thickness and that in clinical symptoms were observed for the fusiform, F(1,59.23)=6.21, *P=*0.016, superior, F(1, 59.34)=4.68, *P=*0.006, and inferior temporal gyrus, F(1,59.64)=4.33, *P=*0.04, where thickness increased with reduced HAM-D scores (Figure 3). In the ACC, a significant interaction of response group was observed where only responders showed relationships between thickness change and symptom improvement, F(1,55.8)=7.46, *P=*0.008 (Figure 5a). Relationships with clinical response in areas showing ECT effects did not interact with the hemisphere.

### Predictive effects

ACC thickness at T2 correlated with overall clinical response to ECT measured at T3, F(1,25)=4.86, *P=*0.037 (Figure 5b). Variations in ACC thickness at baseline trended toward predicting improved response (*P*=0.09). Predictive effects were not observed for any other cortical ROI.

## Discussion

ECT is currently the most effective therapeutic option for treatment-resistant depression; yet its mechanisms remain poorly understood. In this study, we demonstrate that ECT and ECT-related antidepressant response are associated with macrostructural alterations at the cortical level. The results of this study support that neuroplasticity occurs in a network of cortical regions to promote successful therapeutic outcomes. Specifically, our findings show increased cortical thickness across prefrontal regions including the DLPFC, as well as ACC, lateral temporal cortex, temporal pole, parahippocampal gyrus, parietal association and motor cortices bilaterally over the course of an acute ECT treatment series. Follow-up analysis of distinct ROIs, examining the effect of ECT between baseline and the end of treatment, indicates pronounced increases in cortical thickness in the bilateral ACC, ventral paralimbic cortices and temporal association areas. Although the changes in the temporal cortex related to clinical response in general, structural neuroplasticity in the ACC was associated with symptom improvement in patients defined as responders only. ACC thickness measured shortly after the initiation of treatment was indicative of patients' overall clinical response to ECT.

The longitudinal changes in the cortical thickness with ECT were observed across neocortical, limbic and paralimbic cortices—areas constituting functional networks involved in mood regulation, reward and emotion that are similarly implicated in lesion and functional imaging studies of depression.^48, 49, 50, 51, 52, 53^ Imaging research of depression has shown structural and functional abnormalities in the ACC, medial, lateral and ventral prefrontal cortex as well as in the lateral temporal lobe, particularly in patients with severe and more persistent symptoms.^21, 22, 23^ In the current study, both the fusiform and superior temporal cortex showed effects of diagnosis, established by comparing patients and controls at baseline, as well as the effects of ECT. Furthermore, fusiform gyrus, superior and inferior temporal cortices, areas that integrate and evaluate sensory information relevant to emotion and mood processing,^54^ increased in thickness in association with clinical response.

On the basis of functional imaging findings and other empirical data, current theories support that depression-related pathophysiology stems from under-reactive dorsal frontolimbic networks centrally involved in mood regulation as well as from over-reactive ventrolimbic networks, which modulate the hypothalamic–pituitary–adrenal axis and govern emotional responses.^51, 52, 53, 55^ Initial evidence suggests that regions comprising these circuits are affected by treatment.^56, 57, 58^ For example, prior studies suggest that a dysregulation of the prefrontal circuitry, linked with the cognitive control of emotion,^48, 49, 59, 60^ contributes to a diminished capacity to modulate elevated ventrolimbic responses to emotional stimuli.^49, 61, 62, 63, 64^ Abnormal functional and structural connectivity measured from the resting state and diffusion imaging data point to disruptions across several corticolimbic regions.^35, 65, 66^ For example, ECT-mediated neuroplasticity is observed in white matter pathways connecting prefrontal and temporal lobe association regions and show associations with antidepressant response.^66^ In the context of the existing literature, the current results further suggest that ECT-related neuroplasticity is dispersed across both dorsal and ventral corticolimbic circuits.

Whereas significant associations between symptom change and thickness were detected in paralimbic and temporal association areas, these relationships differed in the dorsal ACC between treatment responders and non-responders. The ACC is densely connected with both prefrontal and limbic areas, and contributes to emotional aspects of decision-making and reward as well as to self-referential information. In depression, interactions at the level of the dorsal ACC are proposed to interfere with recruitment of prefrontal regions to affect emotional responses.^67, 68, 69, 70^ We have also recently shown that ECT normalizes resting-state functional connectivity in striatothalamofrontal circuits, specifically between dorsal ACC and the thalamus/basal-ganglia network, and between the ventral striatum and the anterodorsal default-mode network.^35, 71^ Although the subgenual ACC is also widely integrated into models of antidepressant response,^59^ we did not find treatment-related changes in cortical thickness in this region specifically. Our findings support that plasticity in ACC, a major processing hub for information passing from ventral limbic regions to the prefrontal cortex, is particularly relevant to antidepressant response. Notably, our results also indicate that structural variations in the ACC early in the course of treatment may act as a biomarker for predicting overall treatment efficacy.

Neurotrophic factors responsible for the growth, survival and maintenance of neurons are altered in depression^72, 73, 74^ and have been proposed to underlie ECT response.^7, 75^ Consequently, neurotrophic processes affecting the size and/or density of neurons and glia and their connections may account for ECT-induced changes in cortical thickness in dorsal and ventral limbic circuits.^74^ For example, brain-derived neurotrophic factor is reduced in post-mortem samples of depressed patients and is additionally shown to normalize with the antidepressant therapy including ECT.^76^ Neurogenesis, occurring in the hippocampal dentate gyrus and subventricular zone throughout life, is shown to increase with antidepressant treatment^77, 78, 79, 80^ and ECS,^7, 75^ and is influenced by neurotrophic factors.^81, 82^ Some proportion of neural progenitor cells migrate and are integrated into existing functional circuits,^82^ which may have an impact on thickness, at least in proximal entorhinal and paralimbic cortex. However, cell proliferation and subsequent differentiation to endothelial cells, oligodendrocytes and other neuroglia, and glial activation are shown in the cortex after ECS as well as after specific pharmacological treatment (that is, fluoxetine)^10, 83^ (noting that negative findings also exist^84^). Interestingly, one recent study reports markers for neural progenitor cells in the prefrontal cortex after ECS,^14^ implying that neurogenesis may extend to other areas of the cortex. Neocortical areas other than the prefrontal cortex are more rarely investigated in preclinical studies, and thus whether these effects occur in other cortical regions is largely unknown.

Cortical thickness, ranging between 1.5 and 4.5 mm by cytoarchitectural estimates, varies across the cortex and reflects cellular size and density, extracellular features as well as properties of the neuropil, including myelination. Other processes that may influence cortical thickness include synapto-, axio- and dendrogenesis, which, similar to neurogenesis, are regulated by neurotrophins (including brain-derived neurotrophic factor).^85^ As synapto- and dendrogenesis can occur rapidly in all cortical and subcortical areas,^82^ these processes may explain the more acute antidepressant action of ECT. Synaptic plasticity is also regulated by glutamatergic and GABA-ergic neurotransmission as well as by other neurotransmitters such as serotonin,^82^ which may account for common antidepressant mechanisms across different treatment modalities. As significant changes in cortical thickness were observed only after the end of the ECT index, whereas significant change in mood occurred shortly after the initiation of ECT, results suggest that histological factors such as those mentioned above may more directly relate to antidepressant response. However, as effects of ECT were more pronounced when using linear models than when comparing thickness between baseline and the end of the index series, results suggest that microscopic changes in the cortical architecture are temporally linked with macroscopic changes in cortical thickness.

Although changes in cortical thickness across lifespan measured with MRI show similar spatial patterns to those observed in cytoarchitectural studies,^86^ it is not yet possible to determine the exact nature of MRI signal change, which limits interpretation of results. Owing to the naturalistic design of this study, potential effects of anesthesia or placebo effects could not be completely excluded. The failure to include an untreated or treatment-as-usual patient group as a control may also present a limitation of the current study, as it is possible that remission and associated changes in the cortical gray matter may occur spontaneously over time. However, as patients with severe depression eligible for ECT have low rates of spontaneous remission, this potential limitation is considered less likely to have an impact on findings. Here, the current results demonstrate that ECT elicits bilateral changes in cortical thickness across both dorsal and ventral corticolimbic circuits, and variations in thickness relate to treatment response in temporal and paralimbic regions, whereas thickness in the dorsal ACC discriminates treatment responders from non-responders. These findings support the neurotrophic model of antidepressant response and indicate promise for determining individual neural characteristics that may better determine more personalized future treatment approaches.

## Figures and Tables

**Table 1:** Demographic and clinical characteristics

	*Patients with MDD*, N=*29*	*Controls,* N=*29*
Gender (M/F, %F)	11/18 (62.1%)	13/16 (55.2%)
Age, mean (s.d.)	41.0 (13.5)	40.1 (12.4)
		
*Race/ethnicity*		
African American	1	3
Asian	4	3
Hispanic	4	1
White	19	21
Multi-ethnic	1	1
Education (years), mean (s.d.)	15.31 (2.66)	16.97 (2.34)
Dextral/non-dextral (% dextral)^a^	21/8 (72.4%)	25/8 (86.2%)
Unipolar/bipolar diagnosis	24/5	
Age of onset, mean (s.d.)	23.89 (13.57)	
Current episode, mean (s.d.)	2.37 (4.40)	
Lifetime illness, mean (s.d.)	17.15 (11.93)	
Responders/non-responders (%)^b^	18/11 (62.1%)	
Treatments in index, mean (s.d.)	11.241 (3.24)	
RUL treatment, mean (s.d.)	9.3793 (3.28)	

*Mood scales*	*T1*	*T2*	*T3*
HAM-D^c^	26.3 (4.93)	20.5 (6.47)	12.2 (8.26)
MADRS^c^	40.6 (7.57)	32.6 (8.67)	15.6 (11.4)

Abbreviations: C1, control baseline; C2, control follow-up; ECT, electroconvulsive therapy; HAM-D, Hamilton Rating Scale for Depression; MADRS, Montgomery–Åsberg Depression Rating Scale; MDD, major depressive disorder; RUL, right unilateral lead placement; T1, patient baseline; T2, after the second ECT; T3, after the ECT index series.

a Handedness was estimated using the modified Edinburgh Handedness Inventory^38^ with a laterality quotient of <0.7 defined non-dextrals.

b Response defined as >50% improvement in HAM-D scores over the course of treatment.

c Significant effect of ECT (*P*<0.0001).
