# Supplementary material for: Electroconvulsive therapy and structural neuroplasticity in neocortical, limbic and paralimbic cortex
Source: Transl Psychiatry. 2016 Jun 7;6(6):e832–. doi: 10.1038/tp.2016.102 (PMC4931600; doi:10.1038/tp.2016.102)
Supplement: Supplementary Tables [file tp2016102x1.doc]

| **Supplementary Table 1. Summary of statistical tests** | | |
| --- | --- | --- |
| **Measured Effect** | **Test** | **Description** |
| **Diagnosis Group** | GLM | Cross-sectional GLM. Fixed factor: diagnostic group (MDD, control), Dependent Factor: Baseline cortical thickness (vertex-wise, ROI showing sig. treatment effect). Covariates: age, sex. Sig. level: p<.05. |
| **Treatment** | GLMM | Repeated measures GLMM. Fixed factor= Time point (T1, T2, T3), Random factor= subject. Cortical Thickness measurement: vertex-wise, ROI averaged; FDR Corrected, q=.05 |
|  | Paired t-test | Paired cortical thickness measurement (vertex-wise, ROI averaged) for T1 - T2, T2 - T3, T1 - T3. FDR Corrected, q=.05 |
|  | Post-hoc GLMM | Repeated measures GLMM. Fixed factor= Time point (T1, T2, T3), Random factor= subject, lead placement. Cortical Thickness measurement: vertex-wise, ROI averaged; FDR Corrected, q=.05 |
| **Clinical Response** | GLMM | Repeated measures GLMM. Fixed factor= ΔHAMD_T1-T3_, Random factor= subject, hemisphere. Cortical Thickness measure: ROI showing sig. treatment effect. Sig. level: p<.05. |
|  | GLMM | Repeated measures GLMM. Fixed factor= response group, Random factor= subject, hemisphere. Cortical Thickness measure: ROI showing sig. treatment effect. Sig. level: p<.05. |
| **Predictive of Clinical Response** | GLM | Cross-sectional GLM. Fixed factor: cortical thickness at time point (T1, T2)*, Dependent factor: ΔHAMD_T1-T3_, Covariates: age, sex. Sig. level: p<.05. |

*****Within ROIs showing significant effect of treatment.

| **Supplementary Table 2. Paired t-test of clinical response (HAMD)** | | | | | |
| --- | --- | --- | --- | --- | --- |
| **Time Points** | **Mean Difference** | **t-score** | | **df** | **Significance** |
| T1 - T2 | 4.207 | 5.336 | 28 | | 0.00001 |
| T2 - T3 | 7.241 | 6.382 | 28 | | 6.6E-07 |
| T1 - T3 | 11.448 | 8.941 | 28 | | 1.07E-09 |

| **Supplementary Table 3. Desikan ROI- thickness values (mm) for each hemisphere and time point** | | | | | | | | | | |
| --- | --- | --- | --- | --- | --- | --- | --- | --- | --- | --- |
|  | **Left Hemisphere** | | | | **Right Hemisphere** | | | | | **ECT Effect** |
|  | **T1** | **T2** | | **T3** | **T1** | | **T2** | | **T3** |  |
| **Cortical Region** | **Mean (SD)** | | | | **Mean (SD)** | | | | | **p-value** |
| Anterior cingulate | 2.49(0.25) | 2.5(.26) | 2.57(.24) | | 2.41(.16) | 2.40(.20) | | 2.48(.22) | | **.001** |
| Caudal middle frontal | 2.53(.13) | 2.55(.14) | 2.55(.13) | | 2.58(.08) | 2.61(.09) | | 2.59(.11) | | .087 |
| Cuneus | 1.80(.11) | 1.80(.10) | 1.82(.12) | | 1.77(.11) | 1.77(.1) | | 1.77(.11) | | .473 |
| Entorhinal* | 3.43(.28) | 3.41(.25) | 3.5(.24) | | 3.65(.31) | 3.61(.34) | | 3.76(.32) | | **.001** |
| Frontal pole | 2.81(.23) | 2.78(.22) | 2.74(.26) | | 2.73(.23) | 2.71(.25) | | 2.73(.27) | | .355 |
| Fusiform | 2.53(.14) | 2.54(.19) | 2.57(.16) | | 2.58(.14) | 2.58(.14) | | 2.63(.16) | | **.006** |
| Inferior parietal | 2.47(.13) | 2.47(.13) | 2.50(.14) | | 2.52(.11) | 2.51(.12) | | 2.53(.14) | | .119 |
| Inferior temporal | 2.63(.16) | 2.63(.21) | 2.69(.17) | | 2.75(.18) | 2.75(.17) | | 2.82(.18) | | **.002** |
| Insula | 2.87(.18) | 2.88(.18) | 2.92(.17) | | 2.87(.2) | 2.89(.18) | | 2.9(.16) | | .068 |
| Isthmus cingulate | 2.45(.21) | 2.43(.21) | 2.43(.2) | | 2.34(.19) | 2.32(.19) | | 2.35(.21) | | .601 |
| Lateral occipital | 2.21(.11) | 2.2(.08) | 2.21(.11) | | 2.21(.11) | 2.21(.09) | | 2.24(.11) | | .252 |
| Lateral orbitofrontal | 2.68(.18) | 2.68(.18) | 2.70(.19) | | 2.63(.16) | 2.62(.14) | | 2.63(.12) | | .547 |
| Lingual | 1.96(.12) | 1.95(.13) | 1.95(.13) | | 1.94(.12) | 1.95(.13) | | 1.97(.13) | | .689 |
| Medial orbitofrontal | 2.43(.16) | 2.41(.15) | 2.46(.14) | | 2.32(.14) | 2.32(.12) | | 2.33(.13) | | .310 |
| Middle temporal | 2.82(.14) | 2.82(.18) | 2.85(.13) | | 2.88(.17) | 2.89(.15) | | 2.92(.14) | | .041 |
| Paracentral | 2.32(.16) | 2.34(.15) | 2.34(.17) | | 2.36(.16) | 2.38(.16) | | 2.38(.17) | | .160 |
| Parahippocampal | 2.79(.31) | 2.79(.32) | 2.86(.34) | | 2.72(.3) | 2.68(.26) | | 2.76(.28) | | .002 |
| Parsopercularis | 2.59(.14) | 2.59(.15) | 2.61(.16) | | 2.63(.14) | 2.66(.15) | | 2.64(.20) | | .409 |
| Parsorbitalis | 2.77(.23) | 2.75(.2) | 2.82(.21) | | 2.79(.19) | 2.80(.18) | | 2.83(.15) | | .040 |
| Parstriangularis | 2.58(.18) | 2.57(.19) | 2.6(.18) | | 2.55(.15) | 2.56(.16) | | 2.58(.15) | | .141 |
| Pericalcarine | 1.61(.12) | 1.61(.13) | 1.6(.13) | | 1.58(.13) | 1.58(.12) | | 1.59(.12) | | .989 |
| Postcentral | 2.09(.11) | 2.08(.11) | 2.11(.14) | | 2.07(.11) | 2.08(.13) | | 2.08(.14) | | .572 |
| Posterior cingulate | 2.47(.16) | 2.47(.19) | 2.49(.19) | | 2.42(.15) | 2.4(.16) | | 2.41(.14) | | .414 |
| Precentral | 2.56(.11) | 2.58(.12) | 2.56(.18) | | 2.54(.13) | 2.55(.14) | | 2.54(.18) | | .528 |
| Precuneus | 2.35(.13) | 2.36(.12) | 2.37(.16) | | 2.31(.13) | 2.32(.13) | | 2.33(.16) | | .365 |
| Rostral ACC | 2.62(.21) | 2.63(.15) | 2.66(.19) | | 2.53(.24) | 2.5(.18) | | 2.58(.23) | | .025 |
| Rostral middle frontal | 2.38(.12) | 2.38(.12) | 2.4(.13) | | 2.36(.08) | 2.35(.09) | | 2.35(.11) | | .606 |
| Superior frontal | 2.76(.14) | 2.77(.15) | 2.79(.17) | | 2.75(.14) | 2.76(.15) | | 2.76(.15) | | .492 |
| Superior parietal | 2.21(.11) | 2.22(.12) | 2.25(.13) | | 2.21(.1) | 2.23(.12) | | 2.24(.15) | | .066 |
| Superior temporal* | 2.71(.15) | 2.72(.17) | 2.74(.16) | | 2.77(.17) | 2.77(.18) | | 2.82(.17) | | **.002** |
| Supramarginal | 2.54(.15) | 2.53(.16) | 2.56(.17) | | 2.55(.14) | 2.55(.15) | | 2.57(.18) | | .177 |
| Temporal pole | 3.65(.26) | 3.64(.2) | 3.68(.3) | | 3.86(.3) | 3.86(.26) | | 3.92(.3) | | .240 |
| Transverse temporal | 2.23(.24) | 2.23(.26) | 2.26(.25) | | 2.32(.22) | 2.28(.25) | | 2.3(.25) | | .632 |

Bolded p-values are those that survived FDR correction (p-threshold=.0088). * Significant ECT by hemisphere effects. For the entorhinal and superior temporal cortex, a significant time by hemisphere interaction was detected where ECT effects were more pronounced in the right hemisphere.

| **Supplementary Table 4. Desikan ROI- thickness values (mm) controls at each time point and p-values for comparisons between patients and controls at baseline** | | | | | |
| --- | --- | --- | --- | --- | --- |
|  | **Controls T1** | | **Controls T2** | | **Effect of Diagnosis** |
| **Cortical Region** | **Left** | **Right** | **Left** | **Right** | **p-value** |
|  | **Mean (SD)** | | **Mean (SD)** | |  |
| Anterior cingulate | 2.48(.24) | 2.39(.22) | 2.49(.24) | 2.39(.24) | .812 |
| Caudal middle frontal | 2.56(.14) | 2.59(.16) | 2.57(.17) | 2.59(.16) | .484 |
| Cuneus | 1.81(.1) | 1.78(.09) | 1.80(.11) | 1.77(.10) | .586 |
| Entorhinal* | 3.47(.3) | 3.68(.27) | 3.49(.30) | 3.65(.30) | .576 |
| Frontal pole | 2.63(.14) | 2.66(.12) | 2.63(.14) | 2.63(.14) | **.004** |
| Fusiform | 2.47(.12) | 2.51(.13) | 2.47(.13) | 2.51(.15) | .872 |
| Inferior parietal | 2.72(.18) | 2.8(.15) | 2.73(.19) | 2.79(.14) | .125 |
| Inferior temporal | 2.42(.2) | 2.36(.22) | 2.45(.20) | 2.38(.21) | .945 |
| Insula | 2.16(.11) | 2.2(.11) | 2.15(.11) | 2.20(.13) | .333 |
| Isthmus cingulate | 2.71(.16) | 2.67(.18) | 2.71(.16) | 2.66(.19) | .332 |
| Lateral occipital | 1.98(.12) | 1.99(.11) | 1.96(.12) | 1.97(.09) | .239 |
| Lateral orbitofrontal | 2.43(.15) | 2.4(.15) | 2.45(.17) | 2.40(.14) | .261 |
| Lingual | 2.89(.15) | 2.91(.14) | 2.89(.16) | 2.88(.15) | .253 |
| Medial orbitofrontal | 2.75(.4) | 2.76(.21) | 2.77(.38) | 2.76(.21) | .975 |
| Middle temporal | 2.36(.15) | 2.34(.14) | 2.36(.16) | 2.33(.15) | .805 |
| Paracentral | 2.61(.12) | 2.7(.17) | 2.61(.14) | 2.67(.15) | .221 |
| Parahippocampal | 2.86(.19) | 2.84(.21) | 2.85(.22) | 2.80(.19) | .106 |
| Parsopercularis | 2.59(.15) | 2.58(.18) | 2.58(.16) | 2.58(.18) | .503 |
| Parsorbitalis | 1.59(.13) | 1.55(.11) | 1.57(.14) | 1.54(.14) | .324 |
| Parstriangularis | 2.09(.11) | 2.09(.13) | 2.08(.10) | 2.07(.10) | .858 |
| Pericalcarine | 2.5(.13) | 2.49(.14) | 2.51(.16) | 2.47(.17) | .102 |
| Postcentral | 2.56(.15) | 2.54(.17) | 2.58(.14) | 2.54(.15) | .98 |
| Posterior cingulate | 2.37(.15) | 2.35(.13) | 2.37(.16) | 2.35(.15) | .31 |
| Precentral | 2.66(.21) | 2.54(.25) | 2.66(.19) | 2.52(.28) | .673 |
| Precuneus | 2.42(.11) | 2.39(.14) | 2.41(.13) | 2.39(.15) | .225 |
| Rostral ACC | 2.81(.14) | 2.78(.13) | 2.81(.14) | 2.77(.14) | .178 |
| Rostral middle frontal | 2.22(.16) | 2.22(.15) | 2.23(.15) | 2.22(.14) | .762 |
| Superior frontal | 2.79(.18) | 2.85(.16) | 2.80(.18) | 2.85(.16) | **.037** |
| Superior parietal | 2.58(.12) | 2.59(.13) | 2.57(.15) | 2.59(.15) | .201 |
| Superior temporal* | 2.84(.22) | 2.76(.32) | 2.84(.15) | 2.77(.27) | .571 |
| Supramarginal | 3.69(.34) | 3.84(.33) | 3.68(.27) | 3.85(.29) | .943 |
| Temporal pole | 2.25(.19) | 2.28(.24) | 2.21(.17) | 2.32(.27) | .766 |
| Transverse temporal | 2.89(.14) | 2.89(.15) | 2.91(.13) | 2.92(.14) | .657 |

Bolded p-values indicate regions showing significant differences between patients (see Table 2 for means) and controls at baseline, using p<.05.
